# Coarctation of the Aorta and a Parachute Mitral Valve in an Adult With Differential Cyanosis

**DOI:** 10.14740/cr378w

**Published:** 2015-02-09

**Authors:** Jaime Alfonso M. Aherrera, Ma. Teresa B. Abola, Jose Donato A. Magno, Ma. Helga F. Sta. Maria, Lauro L. Abrahan, Richard Henry P. Tiongco, John C. Anonuevo

**Affiliations:** a Section of Cardiology, Department of Medicine, University of the Philippines, Philippine General Hospital, Philippine; b Department of Cardiology, St. Luke’s Medical Center, Global City, Philippines

**Keywords:** Shone’s complex, Coarctation of the aorta, Differential cyanosis

## Abstract

Differential cyanosis may occur in Eisenmenger physiology in the presence of a patent ductus arteriosus (PDA). We present a unique case of a 22-year-old male manifesting as cyanosis of the left upper extremity and both lower extremities, but with preservation of the right upper extremity. Work-up revealed multiple congenital defects, reminiscent of the Shone’s complex. Survival into adulthood is presumed to be due to a PDA, at the expense of a right-to-left shunt. This report highlights the interplay of multiple anomalies documented on echocardiography and MRI, wherein diagnosis was made non-invasively.

## Introduction

Differential cyanosis is defined as cyanosis of the lower extremities with sparing of the upper extremities, as may occur in an Eisenmenger physiology in the presence of a patent ductus arteriosus (PDA) with right-to-left shunt (distal to the left subclavian artery). We present an atypical presentation of differential cyanosis in a young male discovered to have a combination of congenital heart defects. Cyanosis involved the left upper extremity and both lower extremities, with sparing of the right upper extremity. With non-invasive imaging, we found multiple outflow obstructions in the left side of the heart including a moderate mitral stenosis, double-chambered left ventricle, bicuspid aortic valve with severe aortic stenosis (AS), and a coarctation of the aorta. A PDA with a right-to-left shunt was present, which was presumed to alleviate severe heart failure throughout childhood.

## Case Report

**Figure 1:**
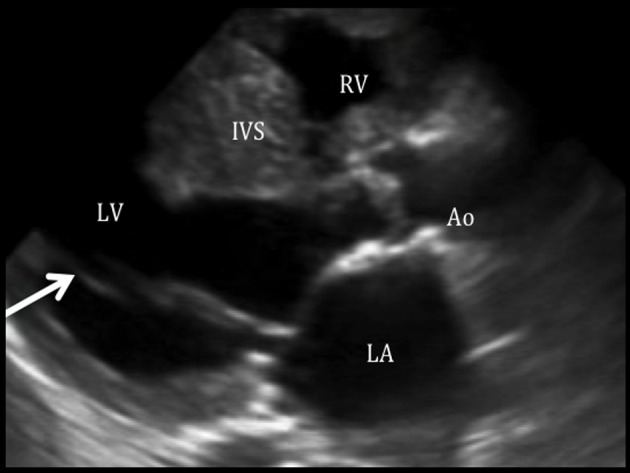
Parachute mitral valve with mitral stenosis on parasternal long axis view. Parasternal long axis view shows both chordae are attached to a single point (arrow), consistent with a parachute mitral valve, giving rise to a mitral stenosis. The left atrium (LA) is dilated with an LA volume index of 45 mL/m^2^. In this view, the aortic valve seems to be thickened. RV: right ventricle; IVS: interventricular septum; LV: left ventricle; Ao: aorta; LA: left atrium.

**Figure 2:**
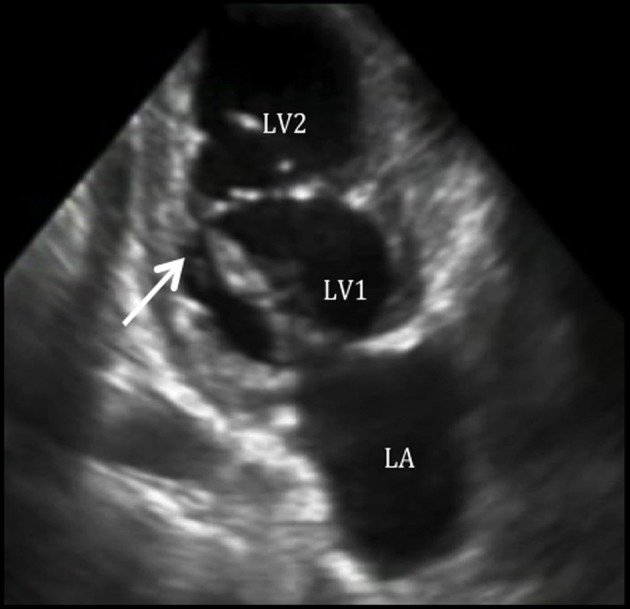
Double chambered left ventricle on two-chamber view. Two-chamber view shows a double chambered LV.

A 22-year-old male sought consult for dyspnea. He was said to have a congenital heart disease after birth presenting with intermittent cyanosis during crying and an alleged “murmur”. During childhood, he had cyanosis during heavy exertion. One year prior to consult, cyanosis would appear more frequently during moderate activity. The cyanosis occurred in his lips, oral mucosa, left hand, and both lower extremities. The right hand was relatively spared during cyanotic spells. Due to decreasing functional capacity and progression of differential cyanosis (limited to the left upper extremity and both lower extremities), he sought consult at our institution. He was a previous methamphetamine user for a year (2012). Blood pressure (BP) was 100/60 mm Hg (right brachial) and 110/70 mm Hg (left brachial). BP on lower extremities was lower at 70 mm Hg palpatory. He had a biventricular heave and a thrill on the third to fourth intercostal space left parasternal border. A grade 4/6 ejection murmur was appreciated on the second intercostal space, left midclavicular side, which radiated to the carotids and the scapula. He had a normal rate and regular rhythm. His abdomen was soft with no bruits on auscultation. He had clubbing on all extremities; however, cyanosis was only noted on his left hand and both feet. Oxygen saturation of his right hand was at 84%, while his left hand and lower extremity saturation ranged from 50% and 70%, respectively. The right radial pulse was slightly weaker compared to the left radial pulse. The femoral, popliteal, and dorsalis pedis pulses were weak as well. A transthoracic echocardiogram revealed eccentric left ventricular hypertrophy with global hypokinesia, a dilated left atrium, right atrium, and right ventricle. He had moderate mitral stenosis from a parachute mitral valve (PMV) (Fig. 1), a double chambered left ventricle (LV) (Fig. 2), AS with a bicuspid aortic valve, coarctation of the aorta, and a right-to-left PDA. The main pulmonary artery was dilated with severe pulmonary hypertension by TR jet. The interatrial and interventricular septa were intact.

**Figure 3:**
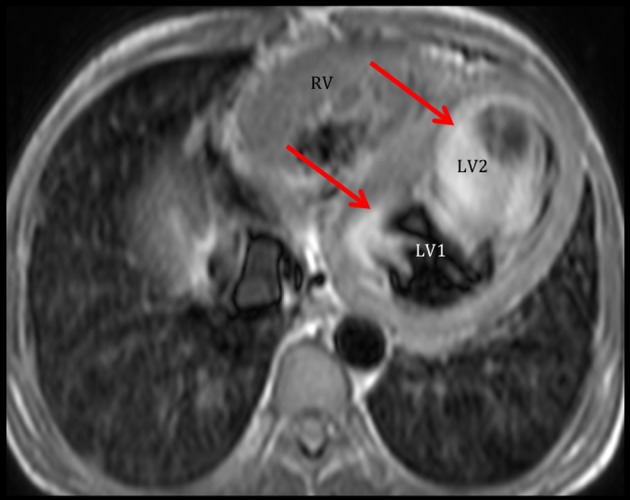
Cardiac MRI demonstrates the double chambered LV. Axial view shows that the LV is partially compartmentalized into two chambers. Note that there is marked flow stasis at the apical part of the LV (LV2) compared to that of the base (LV1). Ejection fraction by cardiac MRI was 15%.

**Figure 4:**
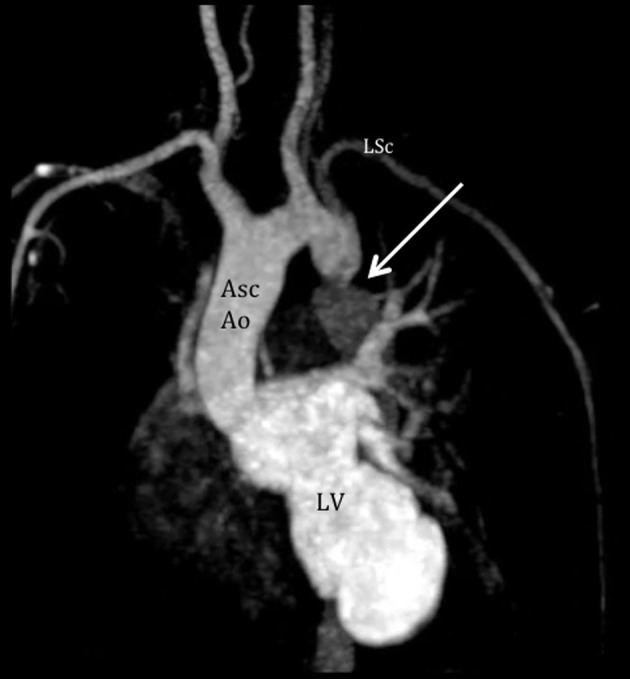
Cardiac MRI demonstrates the coarctation of the aorta. Aortogram shows severe juxtaductal coarctation of the aorta (arrow), 20 mm from the take off of the left subclavian artery (LSc). There is antegrade flow from the LV with significant compromise after the coarctation. Asc Ao: ascending aorta; LSc: left subclavian artery; LV: left ventricle.

**Figure 5:**
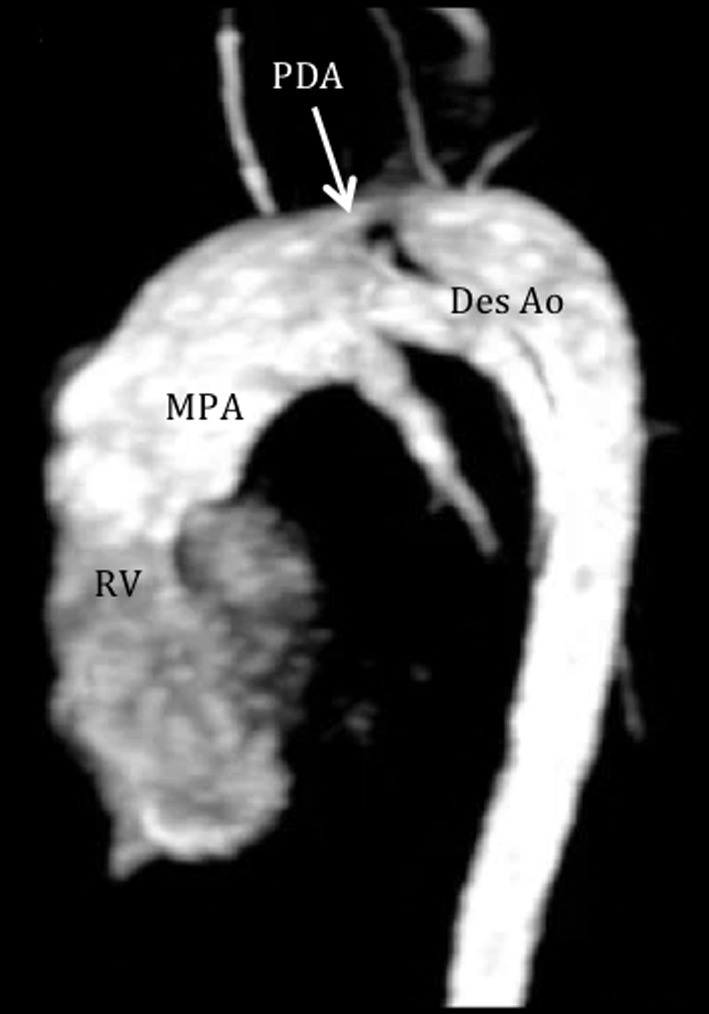
Cardiac MRI demonstrates a patent ductus arteriosus. Pulmonary angiogram shows flow from the pulmonary artery to the descending aorta through a patent ductus (arrow), measuring 8 mm wide and 15 mm in length. RV: right ventricle; MPA: main pulmonary artery; PDA: patent ductus arteriosus; Des Ao: descending aorta.

**Figure 6:**
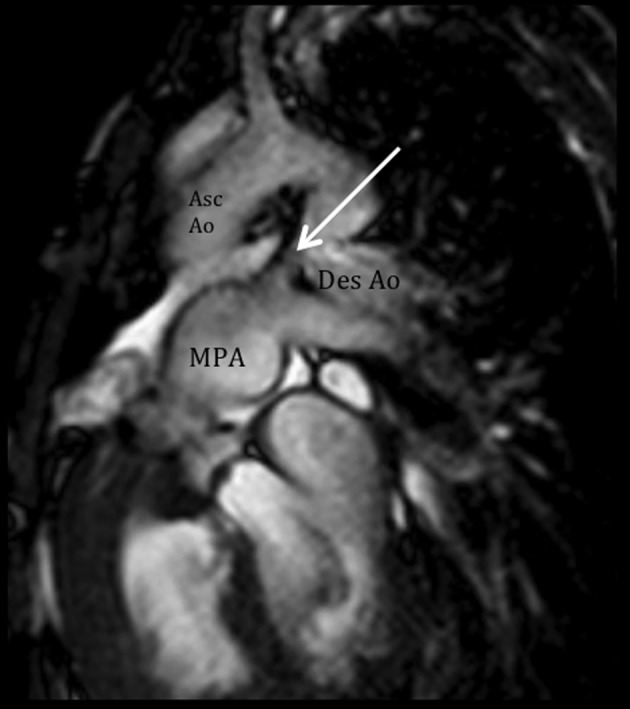
Altered hemodynamics due to multiple anomalies. Cine and velocity flow images show two turbulent jets, consistent with significant right-to-left shunting: 1) an antegrade flow from the dilated pulmonary artery through the ductus (arrow) towards the descending aorta, and 2) a retrograde flow towards the left subclavian artery, predominantly during systole. MPA: main pulmonary artery; Asc Ao: ascending aorta; Des Ao: descending aorta.

A cardiac MRI confirmed the presence of a double chambered LV (Fig. 3), severe coarctation of the aorta after the left subclavian artery with a patent ductus arteriosus at the level of the coarctation (Fig. 4-6). Flow images showed two turbulent jets with right-to-left shunting: 1) an antegrade flow from the pulmonary artery through the duct towards the descending aorta; and 2) a retrograde flow towards the left subclavian artery, explaining the cyanosis of the left upper and both lower extremities. The right upper extremity oxygen saturation was also low, suggesting that this retrograde flow may have extended towards the right subclavian as well. On pulmonary angiogram, there was significant flow towards the descending aorta and left subclavian artery. Other findings were likewise confirmed. Unfortunately, our patient did not consent to cardiac catheterization. An electrocardiogram revealed regular sinus rhythm, left axis deviation, and biventricular and biatrial enlargement. Pertinent laboratory tests included erythrocytosis (hemoglobin of 222 mg/dL) and hypoxemia on the left hand and both lower extremity (PO_2_ 39 mm Hg and 70 mm Hg, respectively).

The assessment was a PDA with a right-to-left shunt and a variant of the Shone’s complex (PMV with mitral stenosis physiology, double chambered LV, bicuspid aortic valve with severe AS, and coarctation of the aorta). Operative repair was offered to our patient to relieve the obstructions. Due to the high-risk nature of any surgical intervention, our patient opted medical palliative management. Our patient eventually succumbed at home during his sleep, presumably due to sudden cardiac death.

## Discussion

Our patient presented with a variety of left-sided obstructions and yet was able to survive into adulthood, presumably due to a PDA with a right-to-left shunt. An entity termed the “Shone syndrome or complex” was first described in 1963. It consists of four obstructive lesions of the left side of the heart, including a parachute-like mitral valve, supravalvular mitral ring, subaortic stenosis, and coarctation of the aorta [1]. Fewer than 100 patients (most in the pediatric population) have been reported in literature and most are detected in childhood as symptoms develop such as dyspnea and signs of reduced cardiac output and acute pulmonary edema [1, 2]. Management is usually surgery during childhood, for definitive repair [3]. Our patient presented with similar features, but did not strictly complete the syndrome. Since it is unusual for these patients to present with minimal symptoms during childhood and survive during adulthood, we believe that a PDA has attenuated pulmonary edema and severe heart failure, allowing our patient to survive to adulthood at the expense of a right-to-left shunt. As seen in the echocardiogram of our patient, the mitral valve chordae are attached to a single point, which is consistent with a PMV, resulting in mitral stenosis (confirmed by cardiac MRI). We believe that this lesion is the initial obstruction *in utero*, predisposing to further constrictions and/or obstructions (double chambered LV, bicuspid aortic valve, and coarctation of the aorta). A PMV is a unifocal attachment of mitral valve chordae independent of the number of papillary muscles, which may result in restricted valve opening [1]. In the Shone complex, it has been noted that the mitral valve obstruction is the most critical problem [4, 5]. In a PMV, the chordae tendinae are underdeveloped causing decreased mobility of the valve leaflets and reducing the size of mitral orifice. Not much is known about its presentation in adults. A review by Hakim et al [6] from 1960 to 2008 included nine cases of PMV in adults. Five had an isolated PMV and four had associated congenital defects. Concomitant cardiac abnormalities are uncommon in adult, because combined complex lesions present early in life with high mortality. Subdivision of the left ventricular cavity is a rare cardiac anomaly, and various classifications have been proposed [7]. Kaski et al [8] described a new variant of a double chambered left ventricle in an 11-year-old girl, similar to our case: a membrane was between the apical and basal areas of the LV, the basal chamber acted as the main chamber which communicated with the LV inflow and outflow tracts, and the apical accessory chamber blood still mixed with blood from the main chamber.

The atypical presentation of differential cyanosis in our patient (pink right hand and blue left hand) may be explained by the two flows noted through a right-to-left PDA on cardiac MRI: 1) one retrograde flow towards the left subclavian artery, explaining the cyanosis of the left hand; and 2) antegrade flow towards the descending aorta, explaining the cyanosis on both lower extremities.

### Conclusion

We have presented a young male with an atypical differential cyanosis diagnosed to have a combination of congenital heart defects, primarily causing multiple obstructions in the left side of the heart. The pathophysiology and hemodynamics of this patient was discussed which was correlated to the clinical manifestations. A similar syndrome (Shone complex) has been described in literature, majority in the pediatric population. Our patient did not fulfill the Shone’s complex because a subvalvar AS and a supravalvular mitral ring were not present. To our knowledge, this is the first reported case that describes this combination of anomalies who survived into adulthood, as most patients with this type of complex heart disease would have poor outcomes during infancy especially if uncorrected. Management of congenital heart diseases in adulthood remains a challenge, and our patient opted palliative care for his complex condition.

## Abbreviations

PDA: patent ductus arteriosus
BP: blood pressure
PMV: parachute mitral valve
LV: left ventricle
AS: aortic stenosis
